# The Dental Aesthetic Index and Dental Health Component of the Index of Orthodontic Treatment Need as Tools in Epidemiological Studies

**DOI:** 10.3390/ijerph8083277

**Published:** 2011-08-09

**Authors:** Chrystiane F. Cardoso, Alexandre F. Drummond, Elisabeth M.B. Lages, Henrique Pretti, Efigênia F. Ferreira, Mauro Henrique N.G. Abreu

**Affiliations:** 1 Department of Community and Preventive Dentistry, Universidade Federal de Minas Gerais, Avenida Antônio Carlos, 6627–ZIP code 31270.901 sala 3304, Belo Horizonte, MG, Brazil; E-Mails: chrys@nwnet.com.br (C.F.C.); efigeniaf@gmail.com (E.F.F.); 2 Department of Paediatric Dentistry and Orthodontics, Universidade Federal de Minas Gerais, Avenida Antônio Carlos, 6627–ZIP Code 31270.901 sala 3304, Belo Horizonte, MG, Brazil; E-Mails: afdorto@googlemail.com (A.F.D.); bethlages@uai.com.br (E.M.B.L.); hpretti@uai.com.br (H.P.)

**Keywords:** indexes, orthodontics, epidemiology

## Abstract

The present study assesses the validity and reproducibility of two occlusal indices for epidemiological studies—the Dental Aesthetic Index (DAI) and the Dental Health Component of the Index of Orthodontic Treatment Need (DHC-IOTN) for the identification of orthodontic treatment needs. The total of 131 study models was examined by an examiner (orthodontic specialist) for the determination of the DAI and DHC-IOTN. Thirty days later, further assessment was performed to determine the reproducibility. The duration of each exam was measured in seconds with a stopwatch. The indices were compared by a panel of three experts in orthodontics to evaluate validity. The intra-examiner reliability evaluation resulted in an intraclass correlation coefficient of 0.89 for the DAI (95% CI = 0.64 to 1.0) and 0.87 for the DHC-IOTN (95% CI = 0.56 to 0.96). The time spent on the evaluation of the DHC-IOTN was less than the time spent on that of the DAI (P < 0.001). The accuracy of the indices, as reflected by the area under the receiver-operating characteristic curve, was 61% for the DAI (95% CI = 51 to 70; p = 0.037) and 67% for the DHC-IOTN (95% CI = 58 to 77; p = 0.001). Both indices presented good reproducibility and validity.

## 1. Introduction

Since the 1960s, considerable effort has been made to develop a valid, reproducible and standardised orthodontic index. Occlusal indices such as the Index of Orthodontic Treatment Need (IOTN) [1], and the Dental Aesthetics Index (DAI) [2] have been developed to rank malocclusion according to the level of treatment need. Occlusal indices can be defined as methods for determining the level of treatment need or the amount of deviation from normal occlusion and can be used for the evaluation of individual patients and populations [3]. Occlusal indices such as the DAI [2] and the IOTN [1] are used to determine the need or priority for orthodontic treatment in epidemiological surveys.

The Dental Aesthetic Index (DAI), adopted by the World Health Organization, evaluates 10 occlusal characteristics: overjet, negative overjet, tooth loss, diastema, anterior open bite, anterior crowding, anterior diastema, width of the anterior irregularities (mandible and maxilla) and antero-posterior spring relationship [2]. The DAI has four stages of malocclusion severity: a score lower than or equal to 25 (no or slight treatment need), a score between 26 and 30 (elective treatment), a score between 31 and 35 (treatment highly desirable) and a score greater than 36 (treatment mandatory) [4]. The IOTN records the need for treatment based on two components: the Dental Health Component (DHC) and the aesthetic component (AC). The DHC-IOTN consists of a hierarchical scale with five levels: level 1 represents little or no need for treatment and level 5 represents a great need for treatment. It evaluates the malocclusion by means of five characteristics: tooth loss, overjet, crossbite, displacement of the contact point, and overbite [1].

Thus, despite having a similar goal, the DAI and the DHC-IOTN exhibit differences that determine their ability to predict malocclusion and the need for orthodontic treatment. Moreover, the IOTN has been described as an index for easy use [1]. In Brazil, the DAI has been used in national epidemiological surveys organised by the Ministry of Health. Other epidemiological surveys, using DAI or IOTN, were carried out in Brazil [5], Spain [6], India [7] and United States [8]. This study aimed to assess the validity and reproducibility of the DAI and the DHC-IOTN in the identification of orthodontic treatment needs.

## 2. Experimental Section

### 2.1. Sample

This study was carried out between July and October 2009. The study involved the assessment of a sample of 131 pairs of dental casts selected randomly from the archive of the Specialization Course in Orthodontics at the Faculty of Dentistry, Universidade Federal de Minas Gerais, Brazil. This archive contains 198 models of oral cavities of all orthodontic patients from Universidade Federal de Minas Gerais. Models in inadequate conditions (with fractures in casts) and models of patients who had received previous orthodontic treatment were not included. The age of participants whose models were included in this study ranged from 12 to 15 years, an age group recommended in studies of occlusal indices by several authors [2,4]. The patients, whose models were evaluated, were at early permanent dentition.

The sample size calculation was performed by considering the 54.3% prevalence of orthodontic treatment need, as measured by the DAI [9], with a confidence interval of 95% and 5% of level of precision. Approval was obtained from the Ethics Committee in Research of Universidade Federal de Minas Gerais (No 0369.0.203.000-09).

### 2.2. Reproducibility, Validity and Time Evaluations

First, to assess reproducibility, 13 models (10% of the total sample set) were examined on two occasions, with an interval of 30 days between examinations, using the two indices proposed in the study. The reproducibility analysis was carried out before the validation analysis.

After the reproducibility assessment, the 131 study models were examined by the researcher, an expert in orthodontics, to assess the ability of both indices to identify orthodontic treatment need. The DAI [2,4] and the DHC-IOTN [1] values were classified according to the specific criteria of each index studied. The recommendations were used to measure the DHC-IOTN in the models [10]. The aesthetic component of the IOTN was not assessed, as it presents poor association with the clinical condition when used in models [11]. The instruments used were those recommended for each index, namely the periodontal probe for the DAI and the proper ruler for the DHC-IOTN.

The gold standard of orthodontic treatment need was determined by three professors who are experts in the area of orthodontics with at least 10 years of clinical experience [12]. They examined the 131 study models separately. Each model was coded as “no need for orthodontic treatment”, “elective orthodontic treatment” or “orthodontic treatment required” based on the clinical evaluation of each one. Where there was disagreement in the assessment of the models, there was a discussion among the researchers to reach a consensus [12–14].

The DAI scores and degrees of treatment need as determined by the DHC-IOTN were regrouped in a dichotomous manner as follows: “without treatment needs” and “in need of treatment”. The DAI scores were dichotomised as “no need for treatment” (DAI ≤ 25) and “in need of treatment” (DAI > 25). The DHC-IOTN is subdivided into three stages of severity according to the need for treatment. Grade 1 (none) and 2 (little) were considered as not requiring treatment, while Grades 3 (moderate need), 4 (great need) and 5 (very great need) were considered as in need of treatment [1] In the same way, the gold standard evaluation was dichotomised as follows: “without treatment needs” and “in need of treatment”. The latter category included “elective orthodontic treatment” and “orthodontic treatment required”. The time needed to evaluate the indices was measured by a digital stopwatch by the same researcher who evaluated the models.

### 2.3. Statistical Analysis

To assess the reproducibility of the original DAI and DHC-IOTN values, the average estimate of the intraclass correlation coefficient for agreement (95% IC) was calculated. The Cohen Kappa coefficient (95% IC) and the Prevalence-adjusted bias-adjusted Kappa (PABAK) [15] were calculated to measure agreement between the dichotomised DAI and DHC-IOTN. An evaluation of the normality of the variable “time” was conducted using the Kolmogorov-Smirnov test. The comparison between time needed to evaluate the indices was done by the Wilcoxon test. The significance level was set at 5% for all analyses. The validation of the indices was done by calculating sensitivity, specificity, positive predictive value, negative predictive value and accuracy (area under the receiving-operating characteristic curve [ROC curve]). An optimum cutoff point for each of the indexes was determined by plotting ROC curves.

## 3. Results and Discussion

### 3.1. Results

The mean (±Standard Deviation) and median DAI values obtained were 35.4 (±10.9) and 33.0, respectively. The minimum and maximum values were 19 and 98. For the ordinal DHC-IOTN, the minimum and maximum values ranged between 1 and 5. The orthodontic treatment need according to the DAI and DHC-IOTN evaluation made by the examiner were presented on Table 1.

The intra-rater reliability assessment resulted in an intraclass correlation coefficient of 0.89 for the DAI (95% IC = 0.64 to 1.0) and 0.87 for the DHC (95% CI = 0.56 to 0.96). Table 2 shows the comparison between the two indices, the Cohen Kappa and PABAK coefficients.

The time spent (in seconds) to assess the DAI and the DHC-IOTN were presented on Table 3. These variables were not normally distributed (Kolmogorov-Smirnov test, *P* < 0.05). The time spent to assess the DHC-IOTN was statistically lower than that for the DAI (Wilcoxon test, *P* < 0.001).

When comparing the two indices with the gold standard (Table 4), less agreement on the overall diagnosis of models examined for treatment needs was observed (47% according to the DAI and 52% according to the DHC-IOTN), with a significant percentage of false positives both for the DAI (41%) and the DHC-IOTN (39%).

**Figure 1.**
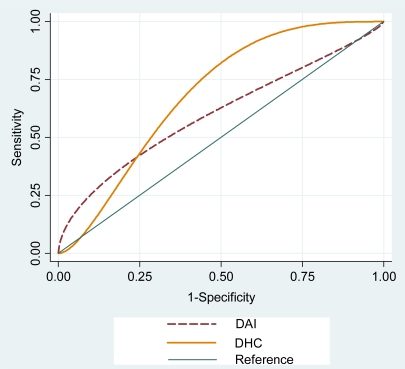
ROC curve for the DAI and DHC, Brazil, 2009.

The accuracy of the indices, as reflected by the ROC curve, was also presented (Figure 1). In the analysis of the validity of the indices (Table 5), both had great sensitivity and very low specificity, indicating a good ability to identify orthodontic treatment need in patients. However, the positive predictive value (PPV) for both indices was low, reducing the certainty of the sensitivity. Otherwise, the specificity is low but the negative predictive value is high. The new cutoff points (DAI=31, and DHC=3), have changed the properties of indexes.

The agreement between assessments of the gold standard and the DHC in three categories (need—borderline—no need) were also fair (Kappa = 0.18 [95% CI = 0.09 to 0.26]).

### 3.2. Discussion

Indices could be considered useful for epidemiological and public health applications when they are reliable and valid. Considering the results presented, the DAI and DHC-IOTN could be considered reliable and validity.

For the sample size calculation of our study we could use the frequency of orthodontic treatment need as measured by the DHC-IOTN or by the DAI. Considering that orthodontic treatment need based on research with orthodontic study models was about 15.0% [16], we opted for the frequency of orthodontic treatment need determined by the DAI [9] because it ensured a larger sample set. The literature have pointed out that it is possible and correct to use dental models in order to validate orthodontic indices [14,17]. Besides, the reliability and agreement between the information obtained clinically and from diagnostic models are high [11]. For these reasons and due to feasibility, we carried out the study using dental models.

High intra-examiner agreement existed between the original DAI and DHC-IOTN values. The examiner was trained and calibrated in the use of the indices before the evaluation sessions, which confirms the need for those steps before an epidemiological survey. This step contributed to the good results. However, the examiner was a specialist in orthodontics, and epidemiological surveys are normally conducted by general dentists, which may point to the need for more previous training. It might be necessary to evaluate the reliability and validity of occlusal indices between general dentists as well. It is important that the indices have a high degree of reproducibility to be useful as a research tool. Despite the lower ICC DHC-IOTN as compared to the DAI value, the confidence intervals are coincident, showing that the reproducibility of both is similar [1,11,12,17,18].

Despite the high percentage of agreement between both indices, the Cohen Kappa could be considered fair. However, considering that the agreement between positive classification (orthodontic treatment need) for DAI and DCH-IOTN was high, Cohen Kappa was artificially low. So, the Prevalence-adjusted bias-adjusted Kappa was calculated and it was considered substantial. So, the DAI and DHC-IOTN measure orthodontic treatment needs in the same way [15].

Most cases showed the need for treatment. This high prevalence is similar to the results of other studies because these validation studies are usually conducted in orthodontics settings, where study models are provided and where most cases for treatment are, due to the need for diagnosis [14,16,17,19].

In validity studies of the occlusal indices, an important factor is the definition of the gold standard. The literature has considered a panel of orthodontists to be the gold standard. This assessment, as defined by several authors [20,21], has been considered the gold standard of the orthodontic treatment needs. However, there are at least two ways to define this panel: using the Likert scale [17] or by consensus [12]. The number of orthodontists in this type of panel has varied from two [12] to eighteen [21]. Our study had a consensus panel of three orthodontists, similar to that of Freer and Freer [12]. It seems necessary to standardise the construction of these panels worldwide to better define the need for orthodontic treatment. It is not easy to infer the effect on the validity statistics of the DHC-IOTN and DAI if the number of specialists participating in the panel were changed. In Brazil, the post-graduate Orthodontic programs vary in content and length of study which may potentially increase the discordance among specialists’ determinations of treatment need.

The comparison with the gold standard has shown an impressive amount of false positives. This is a worrying finding because about 50% of the cases were determined to need treatment based on both indices which a committee of experts in orthodontics had not noticed. In this case, an epidemiological survey using these indices may overestimate the need for treatment in a population. The modification in the cutoff points has decreased the proportion of false-positive and has increased the proportion of false-negative results in both indices. The overall concordance has slightly increased.

In the validity assessment, the DHC-IOTN showed a sensitivity of 100% and DAI, 91%, *i.e.*, the probability of the assessment performed correctly indicate the orthodontic treatment needs is great. Both showed low values of specificity (DAI = 14% and DHC-IOTN = 19%).

In epidemiological surveys, sensitive tests are useful because they prevent people with a problem from being disregarded. Depending on the problem, this can be a complicating factor in finding a solution. Moreover, specific tests are also desirable because they contribute to cost reduction both in the need for subsequent examinations and in the treatment that will be provided. The low specificity is related to a high degree of false positives, which affects the good sensitivity. Thus, it would be desirable to have a balance of these two characteristics, but that did not occur with the DAI and DHC-IOTN indices. Thus, it is necessary to develop an occlusal index that evaluates orthodontic treatment needs more accurately. This development process is not easy and could be done with participation of experts in orthodontics, public health, epidemiology, statistic from all over the world.

The positive predictive value (PPV) for the two indices is low (28 for the DAI and 31 for the DHC-IOTN). Whereas PPV increases with increasing prevalence, this is another deficiency in the validity of these indices. The modification in cutoff points increased the PPV and specificity for the two indices. However, the others properties (sensibility and negative predictive value) have decreased.

The deficiencies observed in the characteristics analysed concerning the validity of a test resulted in the accuracy values (DAI = 0.61 and DHC-IOTN = 0.67). Studies with American [2] or English [1] orthodontists showed better accuracy levels. However, in another study [14], the accuracy of the IOTN was very similar to our results. The validity of an index can depend on the origin of the orthodontic experts who determine as the gold standard [14]. As discussed previously, an expert’s opinion is currently regarded as the best determinant of the treatment need because of the difficulty in using occlusal indices to identify and quantify the objective signs of malocclusion and orthodontic treatment needs [10,21,22]. Therefore, the aggregate decision of orthodontic specialists is generally regarded as the gold standard against which any occlusal index should be validated [20,21]. The different methods of obtaining the gold standard in the validation studies could also explain the different accuracy results for the occlusal indices [12,21].

The time spent for the assessment of the DAI was longer than for the DHC-IOTN. This is probably because only the worst occlusal feature is recorded by the DHC-IOTN [3]. In other words, the identification is made through a hierarchical scale of occlusal anomalies, whereas several occlusal features of space and the teething are recorded to obtain the final DAI score. Reducing the time needed for index application is always important, especially in population studies [22]. Despite not assessing the aesthetic component of the IOTN once we evaluated dental models [11], the inclusion of this component would increase the time spent in evaluation. A disadvantage of DHC-IOTN use is that the proposed ruler for the index is not easily found, whereas the DAI is an index whose instrument for measurement (periodontal probe) is easily accessible.

It is necessary to point out some limitations of this study. The study was conducted with a small group of Brazilian orthodontists, and the sample, although probabilistic, is representative of a single orthodontics service in Brazil. Other studies should be conducted to assess the validity and reproducibility of the DAI and IOTN among Brazilian orthodontists. Although there is little option for orthodontic treatment in public health in Brazil, the choice of a reliable and valid instrument is essential for a correct epidemiological diagnosis. Additionally, the incorporation of subjective evaluation in the epidemiological diagnosis of orthodontic treatment need is absolutely relevant [23,24]. The studied indices are epidemiological tools that aim to assess the degree of treatment need and not make diagnoses or aid in orthodontic planning. The epidemiological indices usually underestimate the studied disease, which has not occurred in this case. Further research in this area is important so that the epidemiological findings can be utilised as a reliable tool for planning and evaluation of public health actions.

## 4. Conclusions

The DHC and the DAI are reproducible and have reasonable accuracy. The biggest problem presented is the high false positive rate compared to the gold standard. The DHC has the advantage of being an index of rapid implementation. The time spent assessing the DAI is greater than that spent assessing the DHC.

## Figures and Tables

**Table 1.** Orthodontic treatment need according DAI and DHC values in Brazil, 2009.

**DAI**	**Frequency (n = 131)**

No need or little need	11%
Elective treatment	28%
Highly desirable treatment	22%
Essential treatment	39%

**IOTN**	

No need	9%
Moderate need	19%
In need of severe treatment	72%

**Table 2.** Comparison of orthodontic treatment need by the DAI and DHC in Brazil, 2009.

**DHC**			

**DAI**	**Need**	**No need**	**Total**
Need	83%	5%	88%
No need	8%	4%	12%
Total	91%	9%	100%

Cohen Kappa coefficient = 0.30 (95% CI = 0.13 to 0.47); PABAK coefficient = 0.74.

**Table 3.** Comparison of time spent (in seconds) to assess DAI and DHC-IOTN in Brazil, 2009.

	**Average time (SD)**	**Median time**	**Range (Minimum-Maximum)**	*P* value *
DAI	118.9 (±37.7)	116.0	46.0–215.0	<0.001
DHC-IOTN	59.5 (±41.9)	47.0	3.0–200.0	

* Wilcoxon test.

**Table 4.** Comparison of orthodontic treatment need between the DAI, DHC and the gold standard in Brazil, 2009.

		**DAI**				**DHC**			
GOLD STANDARD	Cutoff points	**25**		**31**		**2**		**3**	
		Need	No need	Need	No need	Need	No need	Need	No need
	Need	47%	5%	31%	21%	52%	0%	44%	8%
	No need	41%	7%	24%	24%	39%	9%	27%	21%

**Table 5.** Properties of DAI and DHC as compared to the gold standard in Brazil, 2009.

	**DAI (CI95%)**		**DHC (CI95%)**	
Cutoff points	25	31	2	3
Sensitivity	91 (81–96)	56 (44–68)	100 (93–100)	85 (74–92)
Specificity	14 (7–26)	53 (40–67)	19 (10–31)	43 (31–56)
PPV*	28 (24–32)	57 (45–69)	31 (27–34)	62 (54–70)
NPV**	82 (51–95)	53 (40–65)	100 (81–100)	73 (52–87)
Accuracy***	61 (51–70)****		67 (58–77) *****	

* positive predictive value;

** negative predictive value;

*** area under the ROC curve;

**** p = 0.037;

***** p = 0.001.
